# Acute uveal effusion during phacoemulsification with preoperative central serous chorioretinopathy: a case report

**DOI:** 10.1186/s12886-017-0530-3

**Published:** 2017-08-03

**Authors:** Ruiqi Chang, Yu Du, Zhou Peng, Yi Lu, Xiangjia Zhu

**Affiliations:** 1 Department of Ophthalmology, Eye and Ear, Nose, and Throat Hospital, Fudan University, 83 Fenyang Road, Shanghai, 200031 China; 2 Eye Institute, Eye and Ear, Nose, and Throat Hospital, Fudan University, 83 Fenyang Road, Shanghai, 200031 China; 3 Key Laboratory of Myopia, Ministry of Health, 83 Fenyang Road, Shanghai, 200031 China; 4 Key Laboratory of Visual Impairment and Restoration of Shanghai, Fudan University, 83 Fenyang Road, Shanghai, 200031 China

**Keywords:** Uveal effusion, Central serous chorioretinopathy, Case report, Infusion misdirection syndrome, Suprachoroidal hemorrhage, Choroidal hyperperfusion, Hyperpermeability

## Abstract

**Background:**

We report a case of acute uveal effusion during phacoemulsification in an eye with preoperative chronic central serous chorioretinopathy (CSC).

**Case presentation:**

A 55-year-old man with a history of chronic CSC for >18 months presented with bilateral opaque lenses. A preoperative ophthalmic examination showed suspected lenticonus and risky anatomical features, including a thick ciliary body, and anterior rotation of the ciliary process and iris root in both eyes. Optical coherence tomography (OCT) detected CSC in the left eye, but the results of fundus photography and B-scan ultrasonography were unremarkable. The anterior chamber flattened during phacoemulsification. Anterior vitrectomy was quickly performed for suspected infusion misdirection syndrome, and was followed by uneventful surgery. On postoperative day 1, fundus photography, type B ultrasound, and OCT revealed uveal exudation in the macula of the left eye. On postoperative day 50, the patient’s visual acuity recovered to 20/32, and fundus photography, ultrasonography, and OCT revealed complete resolution of the uveal effusion.

**Conclusions:**

This case suggests an association between preoperative CSC and uveal effusion during surgery, because choroidal hyperperfusion and hyperpermeability were present in the patient’s CSC-affected eyes.

## Background

Uveal effusion is the accumulation of fluid in the suprachoroidal layers, with subsequent choroidal and retinal detachment, and is considered a precursor of suprachoroidal hemorrhage and expulsive hemorrhage [1]. It is noteworthy that expulsive hemorrhage can result in permanent vision loss. More than half of all patients with suprachoroidal hemorrhage ultimately experience impaired light perception vision or worse, and <30% of patients achieve a visual acuity of 20/200 [2].

Although uveal effusion is a potential surgical complication of cataract surgery, the technical improvements in cataract surgery have reduced its incidence. Uveal effusion is rarely seen after clear corneal incision cataract surgery [3]. Here, we report acute uveal effusion during clear corneal phacoemulsification and topical anesthesia in an eye with preoperative chronic central serous chorioretinopathy (CSC).

## Case presentation

**Fig. 1:**
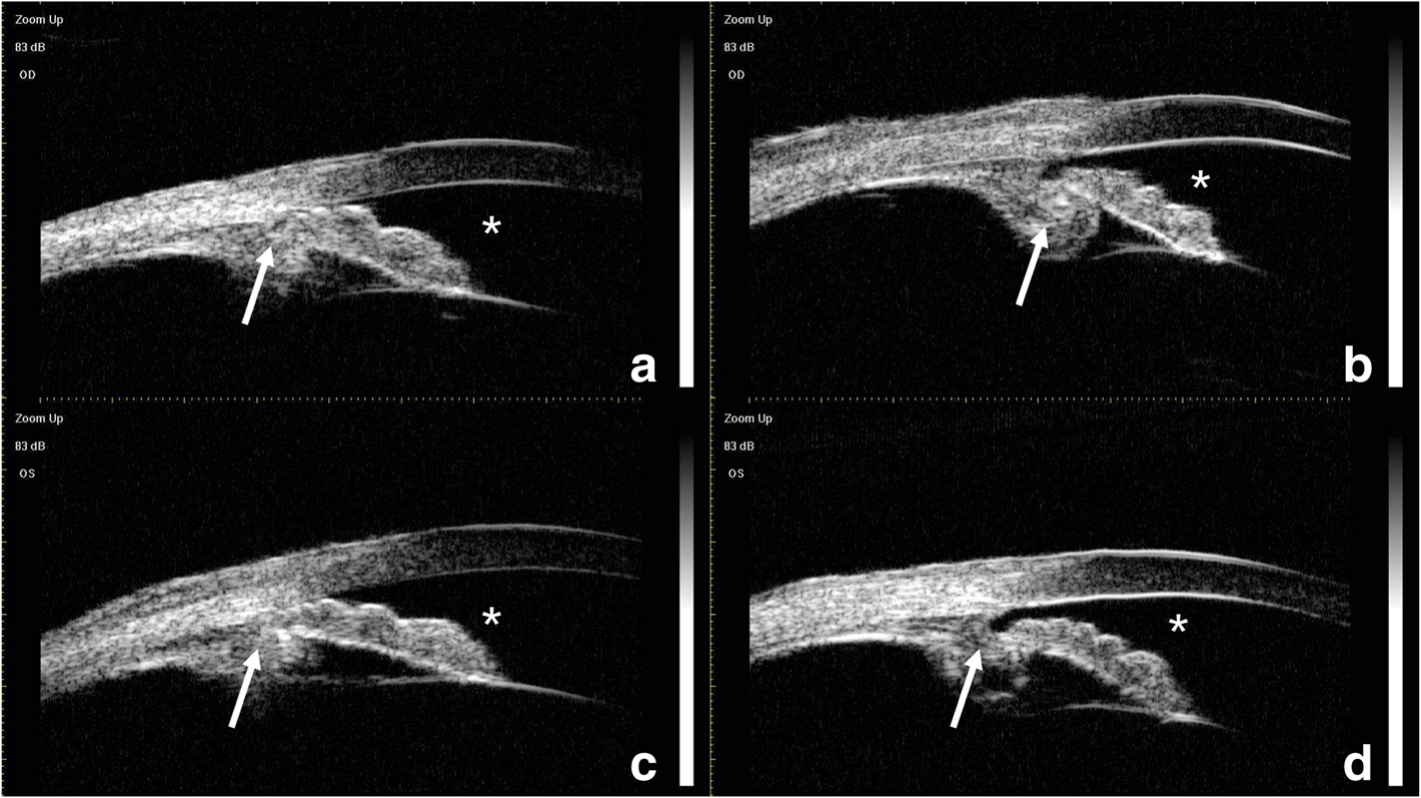
Ultrasound biomicroscopy of the right (**a**, **b**) and left (**c**, **d**) eyes. A shallow anterior chamber (asterisk) with a thick ciliary body and anteriorly rotated ciliary processes and iris root (*arrow*) were detected in both eyes

**Fig. 2:**
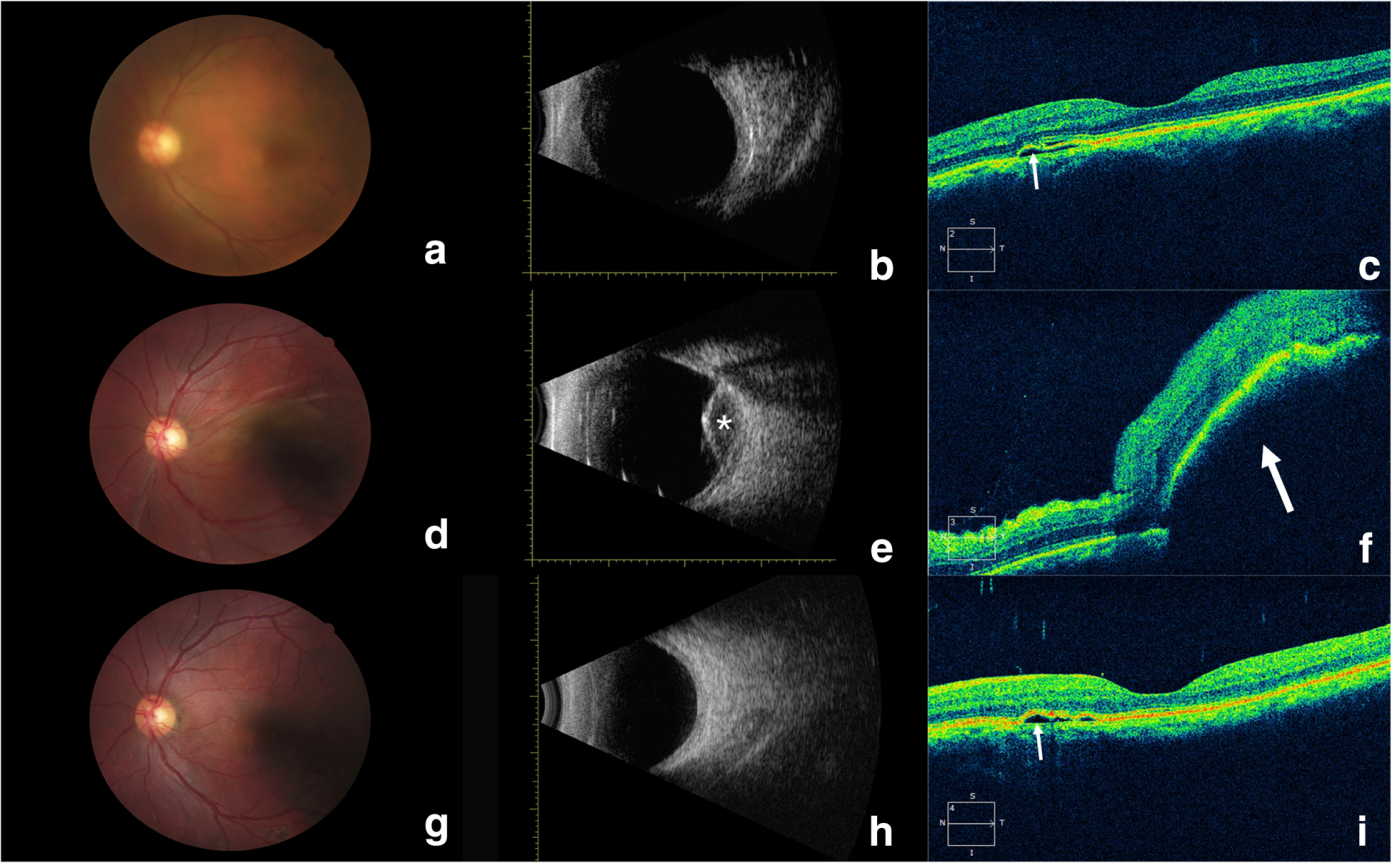
Changes in the eye with uveal effusion. **a**, **b** Preoperative fundus photography (**a**) and type B ultrasonography (**b**) were unremarkable. **c** Preoperative OCT revealed fluid accumulation (*arrow*) beneath the neurosensory retina, resulting in mild serous detachment. **d** Fundus photography on postoperative day 1 revealed suprachoroidal exudation in the macula. **e** Type B ultrasonography on postoperative day 1 revealed serous choroidal detachment, with little blood at the posterior pole (asterisk). **f** OCT on postoperative day 1 revealed a bulging macular choroid with edematous neuroepithelium and subfoveal effusion (*arrow*). (**g**, **h**) Fundus photography (**g**) and type B ultrasonography (**h**) on postoperative day 50 were unremarkable. **i** OCT on postoperative day 50 revealed complete resolution of the uveal effusion and mild serous detachment of CSC (*arrow*)

A 55-year-old man was hospitalized with a predominant complaint of bilateral vision impairment for nearly 15 years. He had a medical history of chronic CSC for >18 months. His uncorrected visual acuity was 20/80 in both eyes. His intraocular pressure (IOP) was 13.2 mmHg in the right eye and 12.8 mmHg in the left eye. A slit-lamp examination revealed a slightly shallow anterior chamber, cortical and posterior subcapsular opacity, and macular pigmentary disorder in both eyes. The axial length was 22.97 mm in the right eye and 22.68 mm in the left eye. Ultrasound biomicroscopy (UBM) showed shallow anterior chambers, with a depth of 2.01 mm in the right eye and 2.03 mm in the left eye, iris bombe, partially occluded slit-like opened angle, zonules in all directions, and a small distance from the lens equator to the ciliary body in both eyes (Fig. 1). The lens thickness, measured using a LENSTAR®, could not be determined in the right eye and was 5.30 mm in the left eye. The results of fundus photography (Fig. 2a), type B ultrasonography (Fig. 2b), and perimetry of the left eye were unremarkable. Fundus fluorescein angiography (FFA) of the left eye showed a hyperfluorescent superofoveal area with several leaking sites. Optical coherence tomography (OCT) revealed serous foveal pigment epithelial detachment on the nasal side, and discontinuity of the inner segment–outer segment junction in the left eye (Fig. 2c). The patient’s medical history included insomnia, but his general physical examinations were normal.

The patient was diagnosed with bilateral cataract, CSC in the left eye, and bilateral suspected lenticonus. He was treated with clear corneal incision phacoemulsification in the left eye under topical anesthesia. The surgery went smoothly until the phacoemulsification probe was exchanged for the irrigation/aspiration probe, when the anterior chamber flattened, resulting in contact between the posterior capsule and the cornea. Attempts to reform the anterior chamber using viscoelastics failed, and the iris prolapsed from the main incision. A vitrectomy lens revealed the presence of a red reflex, unclear fundus details, and the absence of any obvious bump. Therefore, infusion misdirection syndrome was suspected. Dry pars plana anterior vitrectomy using a 25G vitrector was performed, followed by vitrectomy with anterior chamber irrigation soon after the formation of a shallow anterior chamber. The anterior chamber was reformed after anterior vitrectomy, and the rest of the surgery was uneventful. The patient only complained of slight soreness at the end of the vitrectomy. His blood pressure and heart rate were stable throughout surgery.

On postoperative day 1, his visual acuity was finger counting/30 cm in the left eye and his IOP was normal. A slit-lamp examination revealed conjunctival congestion, corneal edema, and a normal depth of the anterior chamber. A fundus examination revealed localized serous choroidal detachment. Fundus photography revealed suprachoroidal exudation in the macula in the left eye (Fig. 2d). Type B ultrasound revealed serous choroidal detachment, with minimal blood at the posterior pole (Fig. 2e). OCT showed a bulging macular choroid, with edematous neuroepithelium and subfoveal effusion (Fig. 2f). During the follow-up period after surgery, the IOP of the left eye was mildly elevated, but remained within the normal range. On postoperative day 50, the patient’s visual acuity had recovered to 20/32. Fundus photography, ultrasonography, and OCT revealed complete resolution of the uveal effusion (Fig. 2g–i).

## Discussion and Conclusions

An association between cataract surgery and uveal effusion was first described by O’Brien in 1935. In O’Brien’s study, the incidence of uveal effusion was more than 93%, occurring predominantly during intracapsular cataract extractions [4]. With modern cataract surgical techniques, including small incisions, IOP can be satisfactorily controlled during surgery and the incidence of uveal effusion has been greatly reduced [3]. Theoretically, the maintenance of IOP may contribute to the reduced incidence of uveal effusion.

Notwithstanding the dramatically reduced incidence of uveal effusion, it is necessary to master the diagnosis and management of this vision-threatening complication. When encountering a sudden shallowing of the anterior chamber during phacoemulsification, it is important to first secure wound closure, and then examine the posterior segment by ophthalmoscopy and transillumination for the differential diagnosis, which includes infusion misdirection syndrome, uveal effusion, and suprachoroidal hemorrhage. One of the main points in the differential diagnosis is choroidal elevation detected by techniques such as ophthalmoscopy. It is noteworthy that, when uveal effusion has just begun, without evidence for choroidal elevation, it is hard to differentiate uveal effusion from infusion misdirection syndrome, as in our case. However, misdiagnosis may lead to inappropriate management and treatment. For infusion misdirection syndrome, which is characterized by a shallow anterior chamber and the absence of choroidal elevation, vitrectomy is recommended, especially if medications, such as 20% mannitol, are ineffective [5]. However, in cases with uveal effusion or suprachoroidal hemorrhage, this procedure is inappropriate and may even aggravate the condition [6]. Type B ultrasonography on postoperative day 1 revealed serous choroidal detachment with minimal blood at the posterior pole, indicating that the correct diagnosis was uveal effusion. Ultrasonography also indicated misdiagnosis of aqueous misdirection syndrome and inappropriate vitrectomy during surgery. The small amount of bloody fluid in the effusion, as detected with type B ultrasonography, was probably caused by the vitrectomy.

The precise mechanism underlying intraoperative uveal effusion is currently unknown. Low IOP might be a contributing factor. Failure to counteract choroidal vessel pressure because of the rapid reduction in IOP during handpiece exchange might cause vessel leakage. Furthermore, unique anatomical features, including a thick ciliary body, and anterior rotation of the ciliary process and iris root, are potential risk factors for uveal effusion [7].

The patient also had a medical history of chronic CSC lasting >18 months and insomnia [8]. CSC is defined as localized and limited serous detachment of the neurosensory retina. Choroidal circulation abnormalities have been observed using various devices. For example, on indocyanine green angiography, CSC presents as a localized delay in arterial filling, followed by choroidal hyperperfusion, such as capillary and venous congestion [9]. Hyperpermeability of the choroidal circulation was also observed with FFA and increased foveal pulsatile choroidal blood flow was detected by laser interferometry in eyes with CSC [9], [10]. All these findings confirm the presence of choroidal perfusion abnormalities, including hyperperfusion and hyperpermeability, in eyes with CSC. With increased intrachoroidal pressure or dilated choroidal vessels, leakage from the choroidal vessels may be followed by uveal effusion. However, on postoperative day 50, OCT revealed mild serous retinal detachment near the fovea, and the location and severity of serous retinal detachment were similar to those detected before surgery. Considering that the patient had a medical history of chronic CSC extending >18 months, preoperative CSC may be a risk factor for intraoperative uveal effusion.

Acute uveal effusion during phacoemulsification has rarely been reported. Here, we have reported acute uveal effusion during clear corneal phacoemulsification under topical anesthesia in an eye with preoperative chronic CSC. This case reveals a potential association between preoperative CSC and uveal effusion during surgery, because choroidal hyperperfusion and hyperpermeability were present in the patient’s CSC-affected eyes.

## Abbreviations

CSC: Central serous chorioretinopathy
FFA: Fluorescein angiography
IOP: Intraocular pressure
OCT: Optical coherence tomography
SRD: Serous retinal detachment
UBM: Ultrasound biomicroscopy
